# *Long noncoding RNA LINC00305* promotes inflammation by activating the AHRR-NF-κB pathway in human monocytes

**DOI:** 10.1038/srep46204

**Published:** 2017-04-10

**Authors:** Dan-Dan Zhang, Wen-Tian Wang, Jian Xiong, Xue-Min Xie, Shen-Shen Cui, Zhi-Guo Zhao, Mulin Jun Li, Zhu-Qin Zhang, De-Long Hao, Xiang Zhao, Yong-Jun Li, Junwen Wang, Hou-Zao Chen, Xiang Lv, De-Pei Liu

**Affiliations:** 1 State Key Laboratory of Medical Molecular Biology, Department of Biochemistry and Molecular Biology, Institute of Basic Medical Sciences, Chinese Academy of Medical Sciences & Peking Union Medical College, Beijing, 100005, P. R. China; 2 Department of Biochemistry, The University of Hong Kong, Hong Kong SAR, P. R. China; 3 Centre for Genomic Sciences, LKS Faculty of Medicine, The University of Hong Kong, Hong Kong, SAR, P. R. China; 4 Department of Vascular Surgery, Beijing Hospital, Beijing 100005, P. R. China; 5 Center for Individualized Medicine, Mayo Clinic Arizona & Department of Biomedical Informatics, Arizona State University, Scottsdale, AZ, 85259, USA; 6 Department of Pathophysiology, Institute of Basic Medical Sciences, Chinese Academy of Medical Sciences & Peking Union Medical College, Beijing, 100005, P. R. China

## Abstract

Accumulating data from genome-wide association studies (GWAS) have provided a collection of novel candidate genes associated with complex diseases, such as atherosclerosis. We identified an atherosclerosis-associated single-nucleotide polymorphism (SNP) located in the intron of the long noncoding RNA (lncRNA) *LINC00305* by searching the GWAS database. Although the function of *LINC00305* is unknown, we found that *LINC00305* expression is enriched in atherosclerotic plaques and monocytes. Overexpression of *LINC00305* promoted the expression of inflammation-associated genes in THP-1 cells and reduced the expression of contractile markers in co-cultured human aortic smooth muscle cells (HASMCs). We showed that overexpression of *LINC00305* activated nuclear factor-kappa beta (NF-κB) and that inhibition of NF-κB abolished *LINC00305*-mediated activation of cytokine expression. Mechanistically, *LINC00305* interacted with lipocalin-1 interacting membrane receptor (LIMR), enhanced the interaction of LIMR and aryl-hydrocarbon receptor repressor (AHRR), and promoted protein expression as well as nuclear localization of AHRR. Moreover, *LINC00305* activated NF-κB exclusively in the presence of LIMR and AHRR. In light of these findings, we propose that *LINC00305* promotes monocyte inflammation by facilitating LIMR and AHRR cooperation and the AHRR activation, which eventually activates NF-κB, thereby inducing HASMC phenotype switching.

Atherosclerotic cardiovascular disease is a major threat to human health and quality of life in modern society^1^. Progression of atherosclerosis is a complex process modulated by multiple factors. Although considerable advances have been made in deciphering the genetic basis of the disease during the past several decades, the mechanisms underlying the development of atherosclerosis have yet to be fully elucidated^2^,^3^,^4^.

Atherosclerosis is considered a chronic vascular inflammatory disorder, and all stages of atherosclerosis development are associated with inflammation. The infiltration of leukocytes and the expression of pro-inflammatory cytokines are among the primary events observed in early atherogenesis. As lesions progress, inflammatory cells secrete pro-inflammatory cytokines and promote tissue inflammation. Biomarkers of inflammation, including C-reactive protein (CRP), are used to predict outcomes in atherosclerosis^5^,^6^,^7^. Monocyte-mediated inflammation plays a critical role in atherogenesis and its complications. Monocytes are the primary inflammatory cells recruited in response to atherosclerosis. Monocytes infiltrate the tunica intima where they differentiate into macrophages. The macrophages engulf modified lipoprotein particles and differentiate into foam cells. Foam cells secrete pro-inflammatory cytokines and amplify local inflammation^7^,^8^. Nuclear factor-kappa beta (NF-κB) is central to the regulation of monocyte inflammation due to its role in coordinating the expression of pro-inflammatory genes, such as those encoding cytokines and chemokines^9^,^10^. NF-κB can be activated by multiple features of atherosclerosis, and the activated form of NF-κB can be found in atherosclerotic plaques. Hence, NF-κB constitutes a promising therapeutic target for limiting the progression of atherosclerosis^11^.

Long noncoding RNAs (lncRNAs) are a class of noncoding RNAs 200 nt or longer in length that have recently been implicated in the regulation of diverse pathophysiological events at all levels of gene regulation^12^. Several lncRNAs, such as *MALAT1*^13^, *ANRIL*^14^ and *LincRNA-p21*^15^, are associated with atherosclerosis and related cellular processes^16^. lncRNAs have also been reported to interact with NF-κB and regulate its activity. The p50-associated COX2 extragenic RNA (PACER) sequesters p50 from genomic DNA to promote the activation of COX-2^17^. The pseudogene lncRNA–Lethe binds to p65 and prevents it from binding to DNA, thereby inhibiting the activation of NF-κB target genes^18^. NF-κB interacting lncRNA (NKILA) blocks the phosphorylation of inhibitor of kappa beta (IkB), thereby inhibiting NF-κB activation^19^.

Genome-wide association studies (GWAS) are an effective strategy for investigating complex diseases, such as atherosclerosis, and have led to the identification of a substantial number of novel susceptible genetic loci associated with various diseases and traits^20^,^21^. These findings have served as valuable resources for uncovering the causal genes and underlying molecular mechanisms that drive corresponding phenotypes^22^. The recent studies evaluating the role of FTO in obesity^23^ and the role of BCL11A in the regulation of globin switching^24^ are representative of the significant developments that have resulted from GWAS follow-up studies. Many loci associated with atherosclerosis and atherosclerosis-related complications have been identified, such as 9p21, and the majority of these represent novel markers^21^. However, few studies combined GWAS result to investigate the long noncoding RNA function in atherosclerosis development.

In this study, we searched the GWAS atherosclerosis database and identified a single nucleotide polymorphism (SNP) located in the intron of *LINC00305*. Functional analysis and additional mechanistic studies demonstrated that *LINC00305* is a novel modulator of NF-κB activity by targeting lipocalin-1 interacting membrane receptor (LIMR) and aryl-hydrocarbon receptor repressor (AHRR), and revealed the role of *LINC00305* in promoting monocytes inflammation and phenotypic switching in human aortic smooth muscle cells (HASMCs), critical steps in the pathogenesis of atherosclerosis.

## Materials and Methods

### Human samples

The human normal arteries were obtained from cadavers, and the atherosclerotic plaques were obtained from surgical resection. Peripheral blood were from both healthy volunteers and atherosclerotic patients, and cord blood samples were from healthy volunteers. All samples were collected with informed consent. Peripheral blood mononuclear cells (PBMC) were separated using Ficoll density gradient and primary monocytes in PBMC were further enriched using the anti-CD14 magnetic beads (Miltenyi). Sample collections and experimental protocols were approved by the Ethical Committee of the Peking Union Medical College & Chinese Academy of Medical Sciences, and carried out in accordance with the approved guidelines (No. 028-2015).

### Bioinformatics analysis

GWAS SNPs associated with atherosclerosis (P-value < 0.0001) were collected from GWASdb database, which include comprehensive GWAS summary statistics from various resources^25^. We then selected candidate variants located in the non-coding regions to identify disease-associated loci. Using an annotation-based effect additive method (GWASrap), we extracted the atherosclerosis GWAS SNPs and identified putative causal variants associated with the original GWAS signals^26^. Briefly, for a given variant, we combined the GWAS summary statistics and the variant functional prediction scores to obtain the final prioritization score. The variant with the highest prioritization score in each high (LD) proxy region of the GWAS SNP were selected to represent the putative causal signal in that locus.

For GO and Heatmap analyses, gene transcriptome profiles of two independent groups of *LINC00305-*overexpressing and control THP-1 cells were detected. The genes increased more than 1.5 times in both group were selected and analyzed by the gene functional classification of DAVID website (https://david.ncifcrf.gov/). The genes involved in inflammation, monocyte activation and chemotaxis (GO: 002544, 002548, 0042177, 0090025) were analyzed with MeV software to show the heat map of the inflammation-relevant genes in the microarray assay.

### Cell culture

The human monocyte cell line THP-1 was grown and maintained in RPIM-1640 medium supplemented with 10% foetal bovine serum (FBS) (Invitrogen) at 37 °C in a 5% (v/v) CO_2_ incubator. HASMCs were purchased from ScienCell (No. 6110, ScienCell) and cultured in Smooth Muscle Cell Medium (SMCM, No. 1101, ScienCell) supplemented with 100 U/ml penicillin, 100 μg/ml streptomycin, smooth muscle cell growth supplement (SMCGS, No. 1152, ScienCell) and 10% FBS. Freshly isolated Human umbilical vein endothelial cells (HUVECs) were cultured in M200 medium, and cells between 3^rd^ to 6^th^ passages were grown into >80% confluence monolayer before used for experiments.

### Gene overexpression

To stably express *LINC00305* in THP-1 cells, *LINC00305* cDNA (NR_027245.1) (Table S1 for the primers) was cloned into the pLV-EGFP-N vector (Inovogen Tech). The empty pLV-EGFP-N vector was used as a negative control.

Lentiviruses with *LINC00305* DNA were prepared according to the supplier’s instructions. Briefly, 25 μg expression vector, 10 μg pLP1, 10 μg pLP2 and 5 μg pLP/VSVG were co-transfected into 293FT packaging cells in a 15-mm dish using Lipofectamine2000 (Invitrogen). The viral supernatant was harvested at 48 h or 72 h after transfection and concentrated by ultracentrifugation. 2 × 10^7^ THP-1 cells were then infected with the viruses at 37 °C for 4 h, and selected using 1 ng/μl puromycin for 2 weeks.

### Western blotting assay

The THP-1 cells were dissolved in radio-immunoprecipitation assay buffer (25 mM Tris-HCl pH 7.6, 150 mmol/l NaCl, 1% NP-40, 1% sodium desoxycholate, and 0.1% sodium dodecyl sulphate (SDS). After a 30-min incubation on ice, samples were sonicated and centrifuged at 4 °C for 30 min. The supernatants were transferred to fresh tubes and protein concentrations were determined using the BCA assay. The samples were boiled with 1 × loading buffer for 10 min before separation by sodium dodecyl sulphate polyacrylamide gel electrophoresis (SDS-PAGE; 20 μg/lane) and transfer to polyvinylidenedifluoride membranes (Millipore). After blocking with 5% bovine serum albumin (BSA), the membranes were incubated with the indicated primary antibodies (Table S3), washed with phosphate-buffered saline (PBS) and incubated with the appropriate horseradish peroxidase-conjugated secondary antibody (Santa Cruz Biotechnology). The immune complexes were visualized using a chemiluminescence reagent.

### Chromatin immunoprecipitation (ChIP) assay

ChIP assays were performed as previously described^27^. A rabbit antibody against p65/RelA was used in the ChIP assay. Control immunoprecipitation reactions were performed using nonimmune normal rabbit IgG. The p65 binding level was calculated by normalizing the ChIP signal with the input signal at the same region. A detailed description of the antibodies used in this study are listed in Table S3, and the primers used to detect cytokine gene promoters in the ChIP assay are listed in Table S4 in the supplemental materials.

### RNA immunoprecipitation (RIP) assay

RIP experiments were performed using the Magna RIP RNA-Binding Protein Immunoprecipitation Kit (Millipore) following the manufacturer’s instructions. A rabbit antibody against the HA-tag was used for the RIP assays. A detailed description of the antibodies used in this study is listed in Table S3, and the primers used to detect *LINC00305* and *GAPDH* in the RIP assay are listed in Table S2 in the Supplemental Materials.

### RNA pull-down assay

RNA pull-down assays were performed as previously described^28^. Briefly, the pEASYblunt simple-*LINC00305* plasmid was used as a template to synthesize the biotinylated *LINC00305* and antisense transcripts. To generate the biotinylated RNA molecules, we performed *in vitro* transcription using the T7 *in vitro* transcription kit (Takara) and the linearized vectors as templates. Ten picomoles of biotinylated RNA were heated at 60 °C for 10 min and slowly cooled to 4 °C. The RNA was combined with 1 mg THP-1 cell lysate in RIP buffer supplemented with 0.1 mg/ml tRNA, 5 mM MgCl_2_ and 80 U SUPERaseIn (Ambion) and incubated at 4 °C for 2 h with gentle rotation. Twenty-five microliters of streptavidin dynabeads (Invitrogen) were added and the reactions were incubated at 4 °C for an additional 2 h. The reactions were then washed thrice with the RIP-supplemented buffer at 4 °C for 5 min each before being boiled in the loading buffer. Finally, the pull-down proteins were separated on SDS-PAGE and visualized using silver staining.

### GST pull-down assay

GST pull-down assays were performed according to the Molecular Cloning Laboratory Manual^29^. Briefly, the coding sequence (CDS) of LIMR was cloned into the pGEX-4T-1 vector. The resulting plasmid was transformed into *E. coli* Transetta to facilitate the expression of recombinant LIMR in the presence of 0.1 mM IPTG at 24 °C for 4 h. Purification of GST-fused recombinant LIMR was performed using Glutathione Sepharose 4B (GE). Ten micrograms purified recombinant LIMR or the control GST were incubated with 1 mg THP-1 cell lysate at 4 °C for 2 h. After the incubation, GST-bound protein complexes were eluted with wash buffer before being boiled in the loading buffer. Finally, the pull-down proteins were separated on SDS-PAGE and visualized using Coomassie brilliant blue staining.

### RNA isolation, quantitative reverse transcription polymerase chain reaction (QRT-PCR) and microarray analyses

Total RNA was isolated using TRIzol reagent (Invitrogen). QRT-PCR was performed according to the manufacturer’s instructions. The standard curves for absolute quantification were obtained by 10-fold serial dilution of pre-quantified template DNA. The primers for the RT-PCR assay are listed in Table S2 of the Supplementary Materials. Transcriptome profiling was performed using the Human Exon 1.0 ST array (Affymetrix) according to the manufacturer’s instruction.

### Reporter assay

The activity of NF-κB was analysed using the dual-luciferase reporter assay (Promega). Briefly, various combinations of pLV-EF1α-LINC00305, pCDNA3.1-LIMR and pCDNA3.1-AHRR were transfected into 293 T cells with pNF-κB-TA-luc (Beyotime). The Renilla luciferase-encoding vector (pRL-TK) was used as the internal control. The luciferase activity (firefly/Renilla) was measured using a Modulus Microplate Multimode Reader and was used as a measure of NF-κB activity.

### Statistical analysis

Quantitative results are expressed as the mean ± standard error of the mean (sem). Comparisons of parameters between 2 groups were analysed using paired/unpaired Student’s t test. Statistical significance was evaluated using the GraphPad Prism 5 software. A p-value less than 0.05 was considered statistically significant.

## Results

### GWAS analysis uncovered *LINC00305* as a putative atherosclerosis-related lncRNA

**Figure 1:**
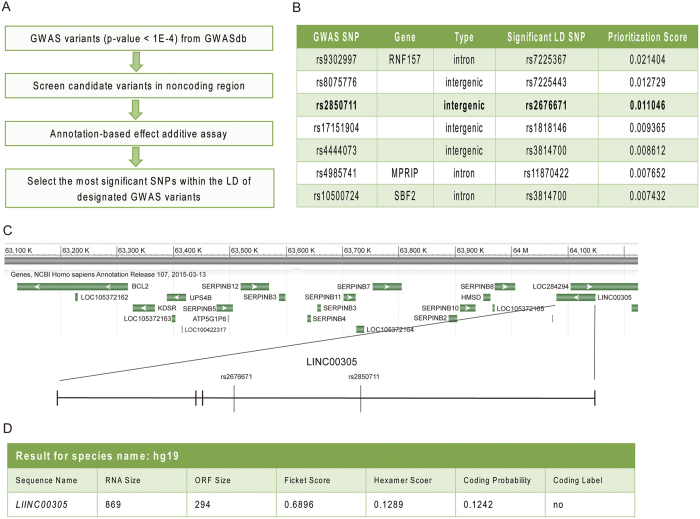
*LINC00305* is a potential atherosclerosis-associated lncRNA. (**A**) Bioinformatic data mining pipeline for collecting and selecting atherosclerosis-associated SNPs^25^,^26^. (**B**) A list of the top rank atherosclerosis-associated SNPs in the polarization assay of A.(**C**) The genomic locus of *LINC00305* and the surrounding regions (from NCBI). The *LINC00305* gene is 69 kb in length and encodes an 869-bp RNA molecule comprising 4 exons. The locations of the 2 atherosclerosis-associated SNPs rs2850711 and rs2676671 within the first intron of *LINC00305* are presented. (**D**) Coding Potential Assessment Tool (CPAT) analysis revealed the low protein-coding potential of *LINC00305*.

To identify novel regulators of atherosclerosis, we searched the public GWASdb database for atherosclerosis-associated SNPs and scored them using an annotation-based effect additive method (GWASrap) (Fig. 1A). Among the SNPs identified, rs2850711 was selected for further analysis based on its high prioritization score. Both this SNP and the putative causal variant rs2676671 in its LD proxy reside in an intergenic region, which was later recognized to encode an lncRNA-*LINC00305* (Refseq id NR_027245, Fig. 1B,C). The analysis therefore implying that *LINC00305* is involved in atherogenesis. *LINC00305* is 69 kb in length and is located in 18q22.1, downstream of the serpin peptidase inhibitor, clade B (SERPINB) genes (Fig. 1C). *LINC00305* exhibited low coding potential according to the Coding Potential Assessment Tool (CPAT)^30^ (Fig. 1D), and no reports are currently available describing the function of *LINC00305*.

### *LINC00305* expression increases in atherosclerosis patients and is primarily associated with monocytes

**Figure 2:**
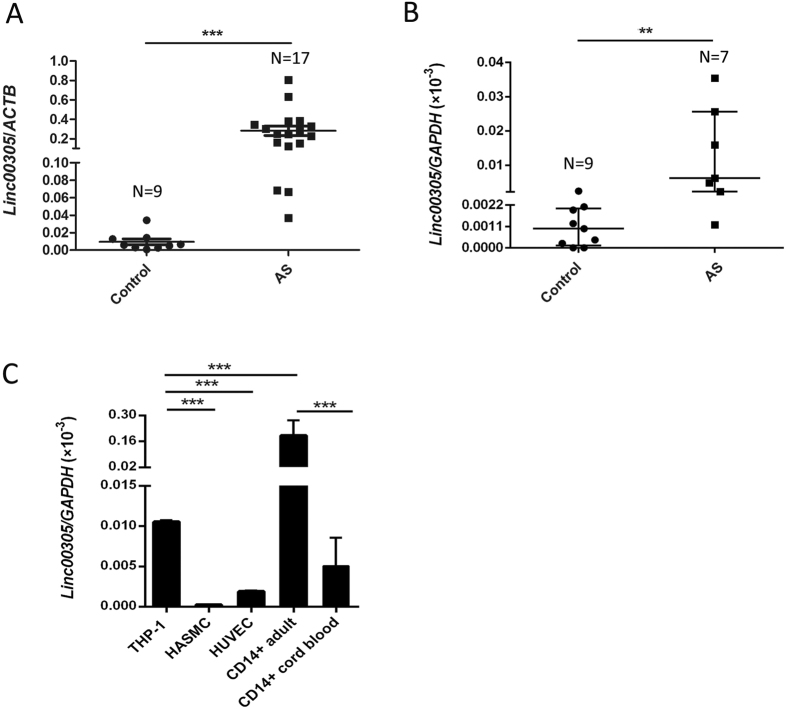
*LINC00305* is upregulated in atherosclerosis and primarily expressed in monocytes. (**A**) Quantitative RT-PCR analysis of *LINC00305* levels in normal human carotid artery samples (Control, N = 9) and carotid atherosclerotic plaques (AS, N = 17). Beta-actin (*ACTB*) is used as the internal control. ***p < 0.001 vs. the indicated group. (**B**,**C**) Quantitative RT-PCR analysis of *LINC00305* levels in (**B**) peripheral blood mononuclear cells (PBMC) from atherosclerosis patients (AS, N = 7) or normal controls (Control, N = 9), and in (**C**) the atherogenesis-associated cell types including the monocytic THP-1 cells, HASMCs, HUVECs, and the magnetically enriched CD14+ monocytes from adult PBMC (N = 5) or umbilical cord blood (N = 4). The level of *LINC00305* was normalized to the *GAPDH* internal control. **p < 0.01, ***p < 0.001 vs. the indicated group.

In light of its potential involvement in atherogenesis, we examined the expression of *LINC00305* in both human normal artery samples and atherosclerotic plaques. *LINC00305* expression was significantly increased in the atherosclerotic plaques compared with the normal artery samples (Fig. 2A, Table S5). Moreover, many other detected human organs also exhibited much lower levels of *LINC00305* expression compared with the atherosclerotic plaques (Fig. S1). Increased *LINC00305* expression was also detected in the peripheral blood mononuclear cells (PBMC) from atherosclerosis patients compared with that from normal controls (Fig. 2B, Fig. S2, Table S6). The specific enrichment of *LINC00305* in atherosclerotic cells and tissue further supports an active role of *LINC00305* in the development of atherosclerosis.

Endothelial cells (ECs), vascular smooth muscle cells (VSMCs) and monocytes are the main cell types found in atherosclerotic plaques^31^. To determine the cellular-specificity of *LINC00305* expression, human umbilical vein endothelial cells (HUVECs), human aortic smooth muscle cells (HASMCs), and monocyte THP-1 cells were collected and examined for *LINC00305* expression levels. *LINC00305* was expressed at substantially increased levels in THP-1 cells compared with HUVEC and HASMC cells. In addition, separated CD14-positive monocytes from PBMC of normal controls showed notably enriched *LINC00305* expression (Fig. 2C, Fig. S2, Table S6). These observations implying that the monocyte is the primary *LINC00305-*expressing cell type. Interestingly, we also showed that *LINC00305* expression in cord blood CD14-positive monocytes was much lower compared with that in the adult counterpart (Fig. 2C, Fig. S2, Table S6). In addition, stimulation of THP-1 cells with lipopolysaccharide (LPS) to induce inflammation upregulates *LINC00305* expression in a time-dependent manner (Fig. S3).

### *LINC00305* promotes monocyte-mediated inflammation

**Figure 3:**
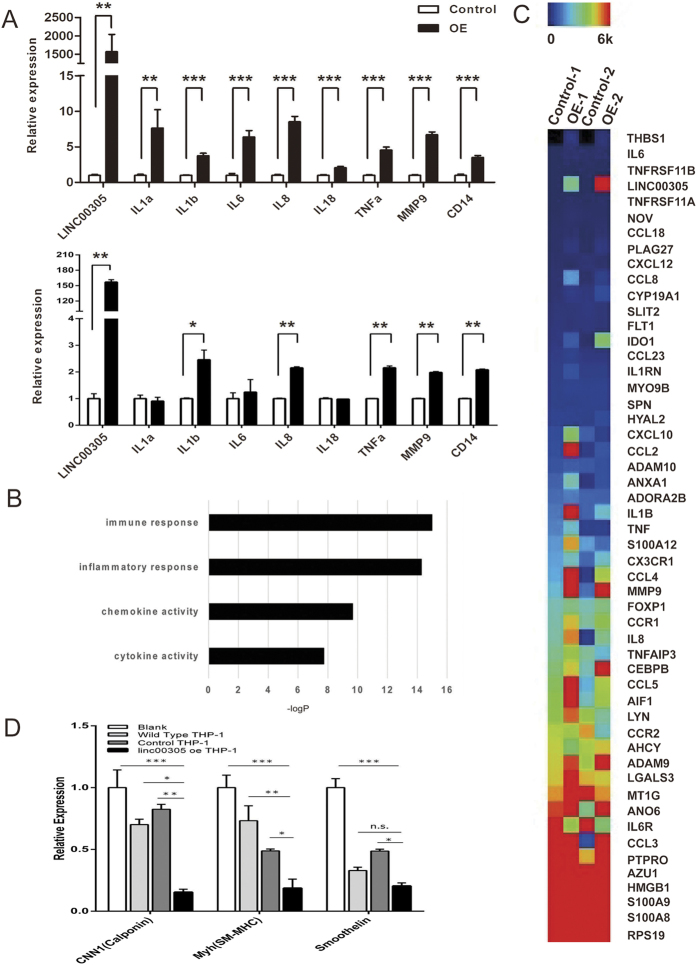
*LINC00305* promotes inflammation in THP-1 cells and phenotype switching in co-cultured HASMCs. (**A**) QRT-PCR analysis of pro-inflammatory gene expression in THP-1 cells stably expressing *LINC00305* (OE) or the control vector (Control). The upper and lower panels show different levels of *LINC00305* overexpression. Gene expression levels in the control groups were assigned a value of 1.0, and *GAPDH* was used as an internal control. Data are presented as the mean ± sem of 3 independent experiments. (**B**,**C**) GO analysis and heat map of the microarray data demonstrated upregulation of inflammation-associated genes in *LINC00305*-overexpressing THP-1 cells. The gene expression profiles of THP-1 cells stably expressing *LINC00305* and the control cells were examined using microarray analysis (Affymetrix, Human Exon 1.0 ST array). Genes upregulated in cells overexpressing *LINC00305* were analysed by GO enrichment analysis (**B**) and the genes involved in inflammation, monocyte activation and chemotaxis (GO:002544, 002548, 0042177, 0090025) were analyzed with MeV software to show the heat map of the inflammation-relevant genes in the microarray assay (**C**). (**D**) QRT-PCR analysis of contractile markers in untreated HASMCs (blank) and in HASMCs co-cultured with wild type THP-1 cells, THP-1 cells stably expressing *LINC00305* (Linc00305 oe THP-1) or the control vector (Control THP-1). The relative mRNA expression levels were normalized to the *GAPDH* internal control. The genes expression levels in the Blank group were assigned a value of 1.0. Data are presented as the mean ± sem of 3 replicate experiments. *p < 0.05, **p < 0.01, ***p < 0.001 vs. the indicated group.

Monocyte-mediated inflammation plays an important role in atherogenesis^8^. To investigate the function of *LINC00305*, THP-1 cells were infected with the *LINC00305*-expressing or the empty control lentiviruses at two different levels. Both levels of *LINC00305* overexpression significantly enhanced the expression of inflammatory genes in THP-1 cells, and the stimulative effect is concentration-dependent (Fig. 3A). However, the expression of *SERPINBs*, genes located adjacent to the *LINC00305* locus were unaffected, indicating that *LINC00305* functions in a trans-regulatory fashion (Fig. S4). To further study the downstream effects of *LINC00305*, the transcriptional profile of *LINC00305-* and empty vector-transfected THP-1 cells were analysed using chip assays (Affymetrix, Human Exon 1.0 ST array). Gene Ontology (GO) analysis demonstrated that *LINC00305-*upregulated genes are enriched for inflammation-associated genes, and similar result was observed in the heat map of inflammation-associated genes in the microarray data, indicating that *LINC00305* promotes inflammation in THP-1 cells (Fig. 3B,C). In atherosclerotic plaques, the cytokines secreted by inflammatory cells promote the shift of VSMCs from a contractile to a synthetic phenotype, an event that contributes to the development of plaques^32^,^33^. To investigate the functional significance of *LINC00305* in atherosclerosis, HASMCs were co-cultured with wild type THP-1, THP-1 cells stably expressing *LINC00305* or the control empty vector. QRT-PCR analysis revealed that co-culture with both wild type and control transfected THP-1 cells decreased the expression of genes associated with the contractile phenotype in HASMCs, and THP-1 cells stably expressing *LINC00305* further down-regulated the expression of these genes (Fig. 3D), suggesting that *LINC00305* overexpression promotes the switch of co-cultured HASMCs from a contractile phenotype to a synthetic phenotype. Together, these results support the hypothesis that *LINC00305* expression plays an important role in the progression of atherosclerosis.

### *LINC00305* promotes inflammation by activating NF-κB

**Figure 4:**
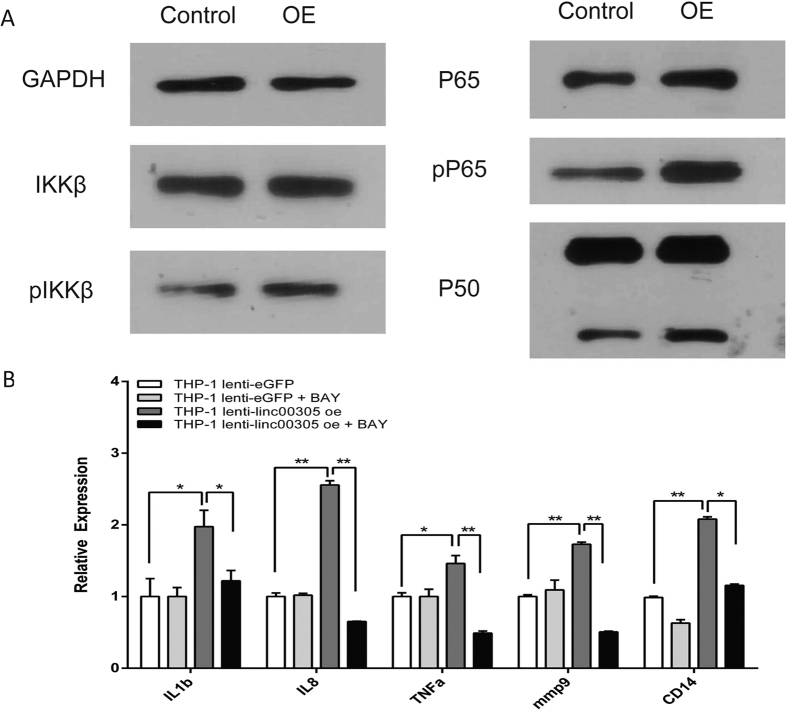
*LINC00305* promotes inflammation by activating NF-κB in THP-1 cells. (**A**) Western blot analysis of the key proteins in the NF-κB pathway (IKKβ, phosphorylated IKKβ, P65, phosphorylated P65 and P50) in THP-1 cells stably expressing *LINC00305* (OE) or the control vector (Control). GAPDH was used as an internal control. (**B**) QRT-PCR analysis of the indicated cytokine genes in THP-1 cells stably expressing the control vector (THP-1 lenti-eGFP) or *LINC00305* (THP-1 lenti-linc00305 oe) treated with DMSO or 10 μM BAY 11-7082 for 30 min. The gene expression levels in the DMSO-treated control group (THP-1 lenti-eGFP) were designated a value of 1.0, and *GAPDH* was used as an internal control. Data are presented as the mean ± sem of 3 independent experiments. *p < 0.05, **p < 0.01 vs. the indicated group.

The NF-κB pathway is essential to the regulation of inflammation^9^,^10^. To investigate the mechanism by which *LINC00305* promotes inflammation in THP-1 cells, we examined the effect of *LINC00305* on the NF-κB pathway. Western blot analysis revealed an increase in IKKβ phosphorylation levels and a significant enhancement of protein levels and phosphorylation of the downstream protein P65 in THP-1 cells stably expressing *LINC00305* (Fig. 4A). Furthermore, immunofluorescence assays demonstrated that P65 translocated to the nucleus (Fig. S5A) and P65 binding to the promoters of the upregulated cytokine genes was shown markedly enhanced in *LINC00305-*overexpressing THP-1 cells (Fig. S5B). Treatment with BAY 11-7082, an inhibitor of NF-κB, abolished the upregulation of cytokine genes observed in THP-1 cells stably expressing *LINC00305* (Fig. 4B). These results demonstrate that *LINC00305* promotes inflammation by activating NF-κB.

### *LINC00305* associates with LIMR and promotes LIMR and AHRR interaction

**Figure 5:**
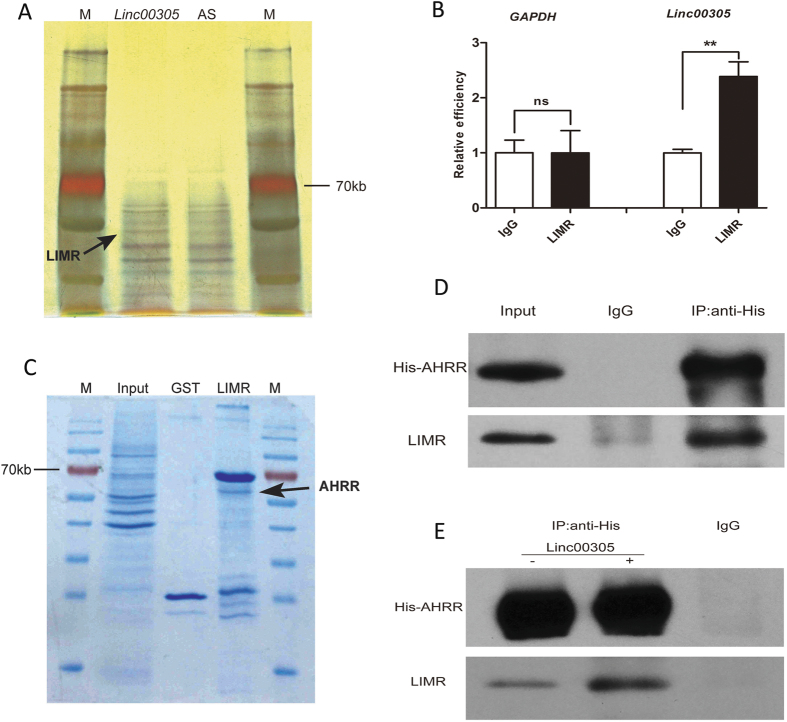
*LINC00305* associates with LIMR and AHRR. (**A**) RNA-pull-down assay to identify *LINC00305* binding proteins in THP-1 cells. The eluted proteins were separated by SDS-PAGE and subjected to silver staining (multiple exposures are presented in Supplementary Figure 6). Antisense RNA to *LINC00305* (AS) was used as a negative control. The black arrow indicates the band representing the *LINC00305-*specific binding protein identified by mass spectrometry as LIMR. (**B**) RIP assay in HeLa cells transfected with *LINC00305* and HA-tagged LIMR. RNA was immunoprecipitated using normal rabbit IgG or the anti-HA antibody. *GAPDH* was used as the negative control. **p < 0.01, ns: no significance vs. the indicated group. (**C**) GST pull-down assay to identify LIMR-interacting proteins in THP-1 cells. The eluted proteins were separated by SDS-PAGE and visualized using Coomassie Brilliant Blue Staining. The black arrow indicates the band representing the LIMR-specific interacting protein identified by mass spectrometry as AHRR. (**D**) Co-IP assay in 293 T cells transfected with LIMR and His-tagged AHRR. Proteins were immunoprecipitated using normal rabbit IgG or anti-His antibody. Input samples and the precipitated proteins were then analysed using anti-His and anti-LIMR antibodies (full length blots are presented in Supplementary Figure 9). (**E**) Co-IP assay in 293 T cells transfected with LIMR and His-tagged AHRR with or without *LINC00305* (full length blots are presented in Supplementary Figure 10).

LncRNAs generally coordinate with protein partners to exert their specific function. To investigate the mechanism by which *LINC00305* activates NF-κB, we sought to identify proteins that directly bind to *LINC00305* using an RNA pull-down assay. The *LINC00305* antisense RNA was used as the negative control. Mass spectrometry analysis of the pull-down results revealed that a band that specifically associated with the sense *LINC00305* RNA represented lipocalin-interacting membrane receptor (LIMR) (Fig. 5A, Fig. S6, Fig. S7A), a 9-pass transmembrane protein that mediates the endocytosis of lipocalin-1 (LCN1)^34^,^35^,^36^. A RIP assay was subsequently performed in Hela cells expressing HA-tagged LIMR using the anti-HA antibody, and the results confirmed that LIMR binds to *LINC00305 in vivo* (Fig. 5B). Moreover, the results of RNA-FISH and immunofluorescence assays demonstrated that *LINC00305* and LIMR co-localize in THP-1 cells (Fig. S8).

So far, no reports are available describing the involvement of LIMR in the process of inflammation, and intracellular partners and downstream signalling pathways associated with LIMR remain unknown. To determine the mechanism by which LIMR potentially mediates the inflammatory effect of *LINC00305* in THP-1 cells, we performed a GST pull-down assay followed by mass spectrometry to identify LIMR-interacting proteins. This approach led to the identification of aryl hydrocarbon receptor repressor (AHRR) as a specific protein partner of LIMR (Fig. 5C, Fig. S7B). Additional immunoprecipitation (IP) assays in 293 T cells transfected with LIMR and His-tagged AHRR confirmed that AHRR and LIMR interact *in vivo* (Fig. 5D, Fig. S9). Moreover, overexpression of *LINC00305* augments the interaction between LIMR and AHRR (Fig. 5E, Fig. S10).

### *LINC00305* activates NF-κB by increasing AHRR protein expression and nuclear localization

**Figure 6:**
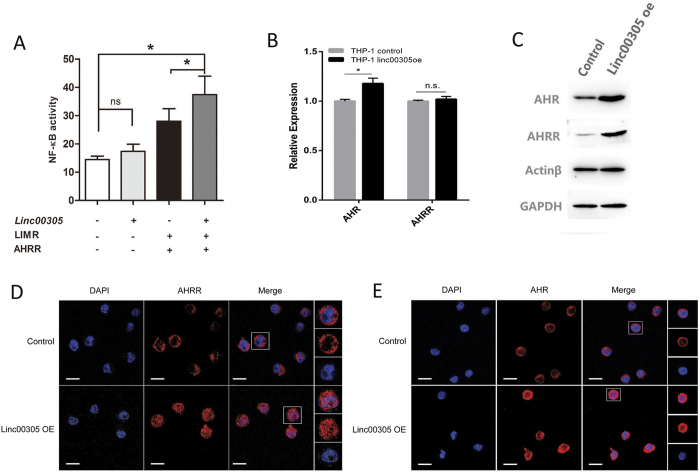
*LINC00305* promotes AHRR protein expression and nuclear localization to activate NF-κB. (**A**) Reporter assay of NF-κB activity in 293 T cells transfected with different combinations of *LINC00305*, LIMR and AHRR. 293 T cells were transfected with *LINC00305*, with or without LIMR- and AHRR-expressing vectors, and the pNF-κB-TA-luc and pRL-TK reporters. The luciferase activity (firefly/Renilla) was measured using a Modulus Microplate Multimode Reader. Data are presented as the mean ± sem of 3 independent experiments. *p < 0.05, ns: no significance vs. the indicated group. (**B**) Real-time RT-PCR analysis of AHR and AHRR expression in THP-1 cells stably expressing *LINC00305* or the control vector. Gene expression levels in the control groups were assigned a value of 1.0, and *GAPDH* was used as an internal control. Data are presented as the mean ± sem of 3 independent experiments. *p < 0.05, ns: no significance vs. the indicated group. (**C**) Western blotting assay of AHR and AHRR expression in THP-1 cells stably expressing *LINC00305* or the control vector. β-Actin and GAPDH were used as the internal controls. (**D**,**E**) Immunofluorescence assays of AHRR (**D**) and AHR (**E**) localization in THP-1 cells stably expressing *LINC00305* or the control vector.

AHRR represses aryl hydrocarbon receptor (Ahr) and Ahr signalling by competitively binding the AHR nuclear translocator (ARNT)^37^. Ahr interacts and cooperates with NF-κB in inflammation regulation, and mainly exhibits an inflammation-suppressive role^38^,^39^,^40^. To evaluate the potential pro-inflammatory role of AHRR, reporter assays were carried out in 293 T cells, and demonstrated that co-transfection of LIMR and AHRR markedly activated NF-κB. Although *LINC00305* alone does not significantly influence NF-κB activity, it significantly activates NF-κB in the presence of LIMR and AHRR (Fig. 6A).

We next examined whether *LINC00305* may affect the expression and nuclear localization of AHRR. Although RNA level of AHRR is unaffected in *LINC00305* overexpressed THP-1 cells, western blotting analysis and immunofluorescence assay revealed notably augmented AHRR protein expression and nuclear translocation in the cells (Fig. 6B–D). AHR expression also mildly increased, at both RNA and protein levels (Fig. 6B,C), but largely remains in the cytoplasm (Fig. 6E). These observations indicated enhanced AHRR signalling pathway upon *LINC00305* overexpression, which then gets dominant in the AHR-AHRR competition for the binding of ARNT and promotes NF-κB activation.

## Discussion

In the present study, we identified a novel inflammation-associated long noncoding RNA *LINC00305* by screening the GWAS atherosclerosis database. An atherosclerosis-associated putative causal SNP was first identified and located to the intron of *LINC00305*, an lncRNA with unknown function. We observed significantly enhanced *LINC00305* expression in atherosclerotic plaques as well as in the PBMCs of atherosclerosis patients, and showed that *LINC00305* primarily expressed in monocytes. *LINC00305* upregulates the expression of genes encoding pro-inflammatory cytokines in THP-1 cells and enhances HASMC phenotypic switching, phenomena that are critical to the development of atherosclerosis. We further demonstrated that *LINC00305* promotes inflammation by interacting with the transmembrane receptor LIMR, augmenting LIMR-AHRR interaction and promoting the protein expression as well as nuclear location of AHRR, which competitively inhibits AHR signalling and promotes NF-κB activation.

### *LINC00305* promotes inflammation by trans-activating the NF-κB pathway

Studies analysing the transcriptome of monocytes or cells stimulated with proinflammatory agents or cytokines have led to the identification of a number of lncRNAs that participate in the activation and the inhibition of inflammation^18^,^41^,^42^,^43^. In the present study, we demonstrate that *LINC00305* is a novel regulator of inflammation. Although many lncRNAs function *in cis* by targeting neighbouring regions, we detected no obvious changes in the expression of genes located near the *LINC00305* locus in cells overexpressing *LINC00305*. Rather, a notable increase in the expression of several pro-inflammatory genes located on different chromosomes was observed, implying that the lncRNA functions as a trans-regulator of gene expression. Previous studies reported that heterogeneous nuclear ribonucleoproteins (hnRNPs)^43^,^44^, the NF-κB pathway proteins p50, RelA and IkB^17^,^18^,^19^ and potentially the PRC2 complex^42^ partner with lncRNAs to mediate inflammation-associated processes. We demonstrated that *LINC00305* stimulates NF-κB signalling, a critical regulator of inflammation^9^,^10^, and that it promotes P65 nuclear localization and enhances P65 binding to its downstream target genes. Moreover, activation of the NF-κB pathway is required for *LINC00305* function.

### *LINC00305* associates with membrane protein LIMR and promotes AHRR nuclear localization

It is generally recognized that lncRNAs regulate gene transcription via interactions with chromatin-modifying proteins and transcription factors^17^,^19^,^45^, and that they potentially regulate mRNA stability and translation in the cytoplasm^46^,^47^. Here, we demonstrated that *LINC00305* localizes to the cytoplasm of THP-1 cells and specifically binds to the transmembrane protein LIMR. In addition, we identified AHRR, the repressor of AHR, as a binding partner of LIMR. We showed that *LINC00305* overexpression enhances the interaction of LIMR with AHRR, and promotes protein expression as well as nuclear localization of AHRR. AHRR was recently reported to promote inflammation in LPS shock^40^, and was shown to increase NF-κB activity upon co-transfected with LIMR in the present study. Although AHR expression was also mildly increased upon *LINC00305* overexpression, it is largely excluded from the cell nucleus and might be a feedback response to the enhanced NF-κB activity. Together, we propose that *LINC00305* promotes inflammation by targeting membrane protein LIMR and modulating its protein interaction as well as downstream signalling. The mechanisms through which *LINC00305* promotes LIMR and AHRR interaction and increases AHRR protein level still requires more investigation.

### *LINC00305* is a novel candidate atherogenesis-associated gene

GWAS analysis provides a powerful strategy for identifying genetic variants associated with complex diseases and phenotypic traits. However, recent study shows that only about 4% of these variants are in the protein coding regions^48^, and the majority of these variants have unknown functions. Follow-up studies are imperative to determine the functional relevance of disease-associated SNPs, especially those located in intergenic regions remote from protein coding genes. The wide variety of lncRNAs discovered in recent years might be involved in the function of some of the intergenic SNPs identified in such studies. A growing body of evidence suggests that lncRNAs might mediate a previously uncharacterized level of regulation in the development of atherosclerosis. *ANRIL*, a gene that resides in the 9p21 locus, is one of the well-studied genes identified by GWAS. *ANRIL* contributes to multiple diseases, including atherosclerosis^49^, potentially by targeting Alu-containing promoters and regulating proliferation, adhesion, apoptosis and senescence in atherosclerotic cells^14^,^49^. Recently, *LincRNA-p21* was reported to be a novel atherosclerosis regulator that influences proliferation and apoptosis of vascular smooth muscle cells and macrophages by enhancing p53 activity^15^. A number of additional lncRNAs participate in the progression of atherosclerosis by regulating atherosclerotic cell proliferation, endothelial cell integrity and lipoprotein expression^50^,^51^,^52^,^53^. Inflammation constitutes another important aspect of the pathology of atherosclerosis. In the present study, we analysed the established GWAS atherosclerosis database and found that *LINC00305*, which contains putative causal atherosclerosis-associated SNP, is a potential regulator of atherosclerosis. *LINC00305* expression is enriched in atherosclerotic plaques as well as in PBMCs of atherosclerosis patients, and is upregulated in LPS-stimulated THP-1 cells. Moreover, *LINC00305* promotes the expression of inflammation-associated genes in THP-1 cells, a critical step in the pathogenesis of atherogenesis^7^,^8^, and promotes the shift of co-cultured HASMCs from a contractile to a synthetic phenotype, another hallmark of atherogenesis. Together, these results indicate a potential role of *LINC00305* in the development of atherosclerosis.

In summary, we identified *LINC00305* as a proinflammatory lncRNA. Our findings indicate that *LINC00305* is a novel lncRNA target that may contribute to advances in the diagnosis of inflammatory diseases and anti-inflammation therapy. The functional role of *LINC00305* in the development of atherosclerosis in patients as well as the mechanisms by which the SNP variations affect *LINC00305* expression remain important questions that merit further investigation.

## Additional Information

**How to cite this article**: Zhang, D.-D. *et al. Long noncoding RNA LINC00305* promotes inflammation by activating the AHRR-NF-κB pathway in human monocytes. *Sci. Rep.* **7**, 46204; doi: 10.1038/srep46204 (2017).

**Publisher's note:** Springer Nature remains neutral with regard to jurisdictional claims in published maps and institutional affiliations.

## Supplementary Material

Supplementary Material
