# Supplementary material for: Long noncoding RNA LINC00305 promotes inflammation by activating the AHRR-NF-κB pathway in human monocytes
Source: Sci Rep. 2017 Apr 10;7:46204. doi: 10.1038/srep46204 (PMC5385552; doi:10.1038/srep46204)
Supplement: Supplementary Material [file srep46204-s1.pdf]

Long noncoding RNA LINC00305 promotes inflammation by activating the AHRR-NF-κB pathway in human monocytes

Dan-Dan Zhang[1#], Wen-Tian Wang[1#], Jian Xiong[1#], Xue-Min Xie[1], Shen-Shen Cui[1], Zhi-Guo Zhao[1], Mulin Jun Li[3,4], Zhu-Qin Zhang[1], De-Long Hao[1], Xiang Zhao[1], Yong-Jun Li[6], Junwen Wang[3,4,5], Hou-Zao Chen[1], Xiang Lv[1,2*], De-Pei Liu[1*]

[1]State Key Laboratory of Medical Molecular Biology, Department of Biochemistry and Molecular Biology, [2]Department of Pathophysiology, Institute of Basic Medical Sciences, Chinese Academy of Medical Sciences & Peking Union Medical College, Beijing, 100005, P. R. China;

[3]Department of Biochemistry, [4]Centre for Genomic Sciences, LKS Faculty of Medicine, The University of Hong Kong, Hong Kong SAR, P. R. China;

[5]Center for Individualized Medicine, Mayo Clinic Arizona & Department of Biomedical Informatics, Arizona State University, Scottsdale, AZ, 85259, USA.

[6]Department of Vascular Surgery, Beijing Hospital, Beijing 100005，P. R. China

[#] These authors contributed equally to the work

[*]**Corresponding authors:** De-Pei Liu, Tel.: (8610) 69156415, Fax: (8610) 65105093, E-mail: liudp@pumc.edu.cn; Xiang Lv, Tel.: (8610) 69156415, Fax: (8610) 65105093, E-mail: lvxiang@pumc.edu.cn

Supplementary Table 1. The primers used for vector construction.

Linc00305	forward	CCGGAATTCAATGATGGACTCTGATTTCCAC
	reverse	GCTCTAGAAAAGTTAAACAAATGTTTAATAGGA
LIMR	forward	GAATTCGCCACCATGGAAGCACCTGACTACG
	reverse	TCTAGATCACTGGTGCTGGGTCTT
AHRR	forward	CCGGAATTCGCCACCATGCCGAGGACGATGATCC
	reverse	TGCTCTAGACTATGGCAGGAATGTGCACC

Supplementary Table 2. The primers used in RT-qPCR and RIP assays.

Linc00305	forward	TCAGCAGCCTTCTGGTTTATCA
	reverse	TCCTTGCTTCCTTCAGGTCTCT
GAPDH	forward	CATGAGAAGTATGACAACAGCCT
	reverse	AGTCCTTCCACGATACCAAAGT
ACTB	forward	CACCCAGCACAATGAAGATC
	reverse	GTCATAGTCCGCCTAGAAGC
IL1α	forward	GGTTGAGTTTAAGCCAATCCATC
	reverse	CACATTGCTCAGGAAGCTAAAAG
IL1β	forward	GGGACAGGATATGGAGCAACA
	reverse	TATCATCTTTCAACACGCAGGAC
IL6	forward	ACTCACCTCTTCAGAACGAATTG
	reverse	CCATCTTTGGAAGGTTCAGGTTG
IL8	forward	ACTGAGAGTGATTGAGAGTGGAC
	reverse	AACCCTCTGCACCCAGTTTTC
IL18	forward	TCTTCATTGACCAAGGAAATCGG
	reverse	TCCGGGGTGCATTATCTCTAC
TNFα	forward	CCTCTCTCTAATCAGCCCTCTG
	reverse	GAGGACCTGGGAGTAGATGAG
MMP9	forward	CTGGGCAGATTCCAAACCTT
	reverse	CGGCAAGTCTTCCGAGTAGTT
CD14	forward	TGAGCTGGACGATGAAGATTTC
	reverse	ACGCGCTTTAGAAACGGCT
Calponin	forward	GTCAACCCAAAATTGGCACCA
	reverse	ACCTTGTTTCCTTTCGTCTTCG
SM-MHC	forward	CATCTACTCGGAGAAGATCGTCG
	reverse	CGCCTGTGCATAGAATGGACT
Smoothelin	forward	GGGATCGTGTCCACAAGTTCA
	reverse	GCTACTCCTCGTTGCTCCTT
SERPINB2	forward	TTAATTCCTGGGTCAAGACTCAAAC
	reverse	AGTAGACAGCATTCACCAGGACC
SERPINB4	forward	CAGCATTAGGGATGGTCCTCTTA

	reverse	TGCAGCTTTTTCTGTGGTGTTC
SERPINB8	forward	AATGACTGGGTGTCGGAAAAAA
	reverse	AACTGAGCCTTCCACTTCCCTT
SERPINB10	forward	GCCAATGCGATATATGGAGAGA
	reverse	CCACAAAGTTAACAGGCTGAGG
VPS4B	forward	CCTCTGATCTTGTTTCTAAGTGGCT
	reverse	CATCAATGAAGATAATGGAGGGCT
KDSR	forward	TCTATTAATGACAAACAGGTGGTGC
	reverse	ACCCAGTTTCTCCTGTGCTTGT
Bcl2	forward	GGTGGGGTCATGTGTGTGG
	reverse	CGGTTCAGGTACTCAGTCATCC

Supplementary Table 3. Antibodies used in western blot, ChIP, RIP, IP and immunostaining assays.

Protein	Producer	Catalog number	Origin
IKKβ	Abcam	32135	Rabbit
phosphorylated IKKβ	Abcam	59195	Rabbit
P65	Abcam	7970	Rabbit
phosphorylated P65	Abcam	86299	Rabbit
P50	Abcam	7971	Rabbit
LIMR	Abcam	103458	Rabbit
AHRR	Abcam	108518	Rabbit
AHR	Abcam	153744	Rabbit
His	Cell Signaling Technology	12698	Rabbit
HA	Abcam	9110	Rabbit
GAPDH	Cell Signaling Technology	5174	Mouse
IgG	Santa Cruz Biotechnology	sc-2027	Rabbit

Supplementary Table 4. Primers used in ChIP assay.

IL1α promoters	forward	TACTGAATGATAGCACGTTTGAGG
	reverse	AGTCTCTTATCACCACCAACACCA
IL1β promoters	forward	GTGTGTCTTCCACTTTGTCCCA
	reverse	AATCGTTGTGCAGTTGATGTCC
IL6 promoters	forward	AACTTCGTGCATGACTTCAGCTT
	reverse	AGCAGAACCACTCTTCCTTTACTTT
IL8 promoters	forward	ATCTTGTTCTAACACCTGCCACTC
	reverse	CTCCACAATTTGGTGAATTATCAA
IL18 promoters	forward	GGGGAAGTCCTGAATGAGGTTAT
	reverse	CCTGGTCACACTTCAGCACAA
TNFα promoters	forward	CATTATGAGTCTCCGGGTCAGA

	reverse	GGCTTGAGGCCTCAGGAAA

Supplementary Table 5. General information of the enrolled atherosclerosis patients (AS, N=17) and normal controls (N=9) who provide the atherosclerotic plaques or artery samples for the RT-PCR analysis of *LINC00305* expression in Figure 2A.

Gender	Age	Diagnosis
Male	87	Normal
Male	61	Normal
Male	73	Normal
Male	22	Normal
Male	47	Normal
Male	57	Normal
Male	64	Normal
Female	71	Normal
Female	73	Normal
Male	57	AS
Male	64	AS
Male	59	AS
Male	53	AS
Male	54	AS
Male	71	AS
Female	74	AS
Female	74	AS
Female	73	AS
Male	62	AS
Male	59	AS
Male	60	AS
Male	59	AS
Male	64	AS
Male	51	AS
Male	56	AS
Male	61	AS

Supplementary Table 6. General information of the atherosclerosis patients and normal controls who provide blood samples for the RT-PCR analysis of *LINC00305* expression in the peripheral blood mononuclear cells (PBMC) (AS, N=7; normal control, N=9) or in the magnetically enriched CD14+ monocytes (normal, N=5) in Figure 2B-C.

Gender	Age	Diagnosis
Male	68	AS

Male	60	AS
Male	40	AS
Male	37	AS
Male	62	AS
Male	71	AS
Male	54	AS
Male	35	Normal
Male	58	Normal
Male	37	Normal
Male	31	Normal
Male	32	Normal
Male	62	Normal
Male	36	Normal
Male	33	Normal
Male	62	Normal
Male (CD14)	37	Normal
Male (CD14)	42	Normal
Male (CD14)	46	Normal
Male (CD14)	53	Normal
Male (CD14)	53	Normal

Figure S1

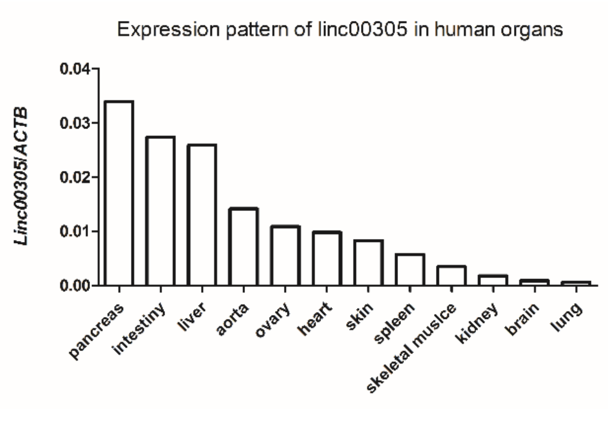

Supplementary Figure 1. The expression of *LINC00305* in different human organs. Quantitative RT-PCR analysis of *LINC00305* in various human organs showed lower expression compare to that in the atherosclerotic plaques (Fig. 2A). The relative mRNA expression levels of mRNA were normalized to the *ACTB* internal control.

Figure S2

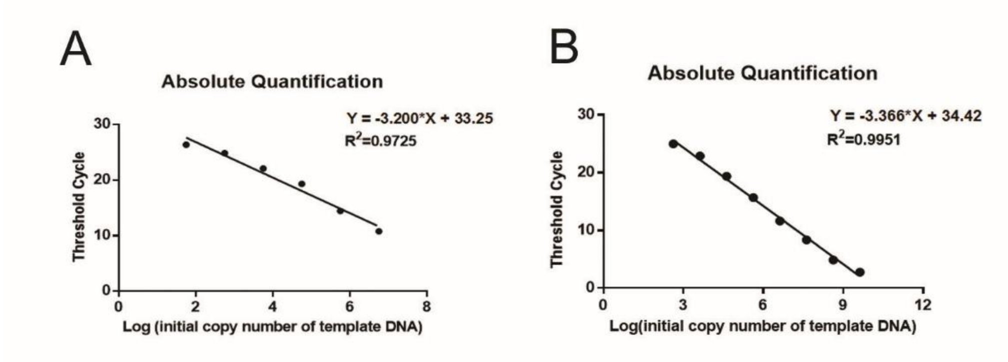

Supplementary Figure 2. Standard curves for the Real-Time RT-PCR quantification of (A) human *GAPDH* and (B) human *LINC00305* expression.

Figure S3

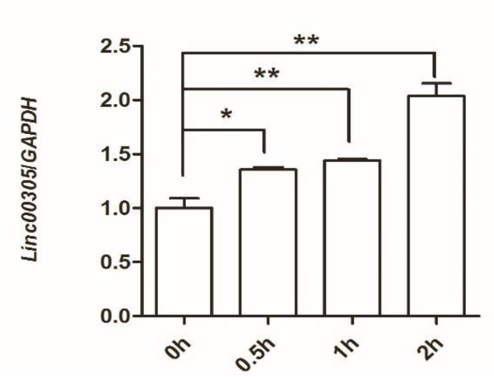

Supplementary Figure 3. *LINC00305* is upregulated in LPS-stimulated THP-1 cells. *LINC00305* expression in THP-1 cells stimulated with 0.1 μg/ml LPS for the indicated periods of time was detected by real-time RT-PCR. *GAPDH* is used as the internal control, and the level of *LINC00305* expression in unstimulated THP-1 cells was set as “1”. *p<0.05, **p<0.01 vs. the indicated group.

Figure S4

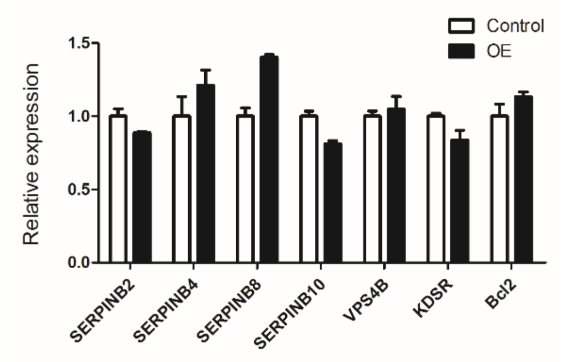

Supplementary Figure 4. Overexpression of *LINC00305* does not significantly affect the transcription of neighboring genes. QRT-PCR analysis of genes adjacent to the *LINC00305* locus in THP-1 cells stably expressing *LINC00305* (OE) or the control vector (Control) (Refs to Fig.3A upper panel for the degree of overexpression). Some neighboring genes are undetectable in the cells and are not shown here. Gene expression levels in the control group were designated a value of 1.0, and *GAPDH* was used as an internal control. Data are presented as the mean ± sem of 3 independent experiments.

Figure. S5

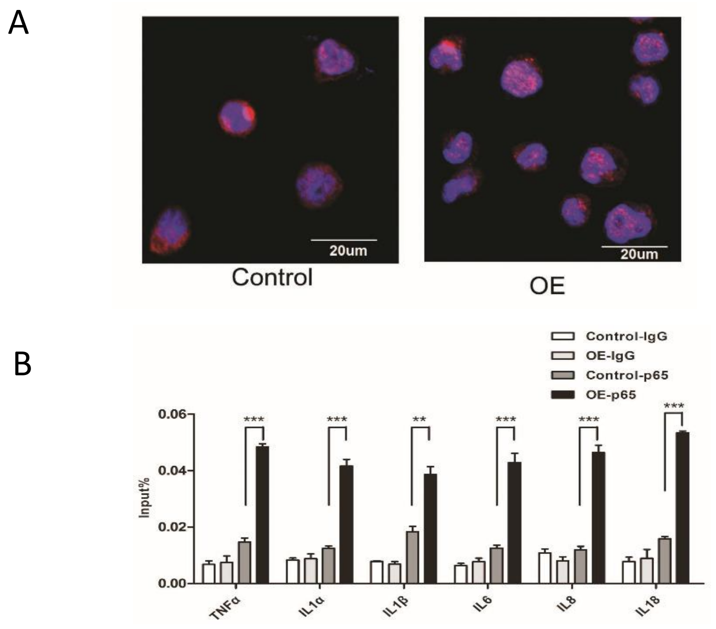

Supplementary Figure 5. *LINC00305* promotes inflammation by activating NF-κB in THP-1 cells. (A) Representative images of immunofluorescence assays evaluating p65 localization in THP-1 cells stably expressing *LINC00305* (OE) or the control vector (Control). （**B**）Chromatin immunoprecipitation (ChIP) assays evaluating p65 binding to the promoters of the indicated cytokine genes in THP-1 cells stably expressing *LINC00305* (OE) or the control vector (Control). Data are presented as the mean ± sem of 3 replicate experiments. **p<0.01, ***p<0.001 vs. the indicated group.

Figure. S6

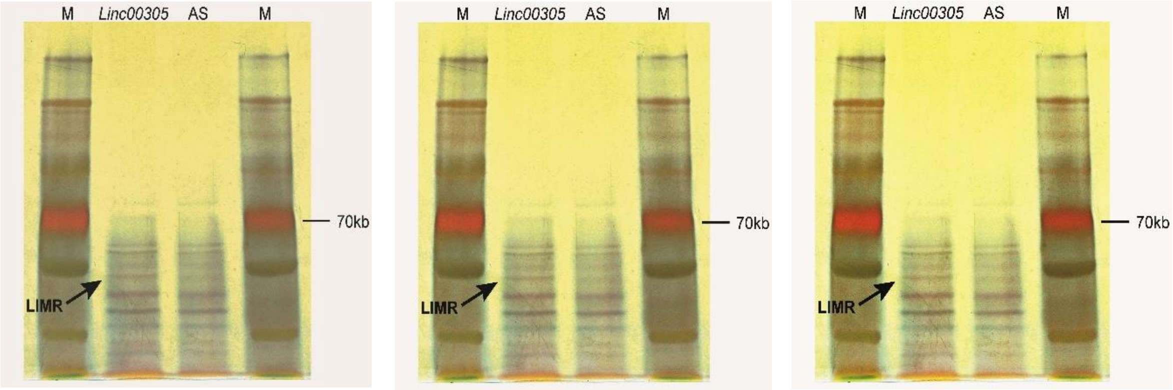

Supplementary Figure 6. Different levels of exposure of the silver staining in Figure 5A. Increased level of contrast is used from the left to the right**.**

Figure. S7

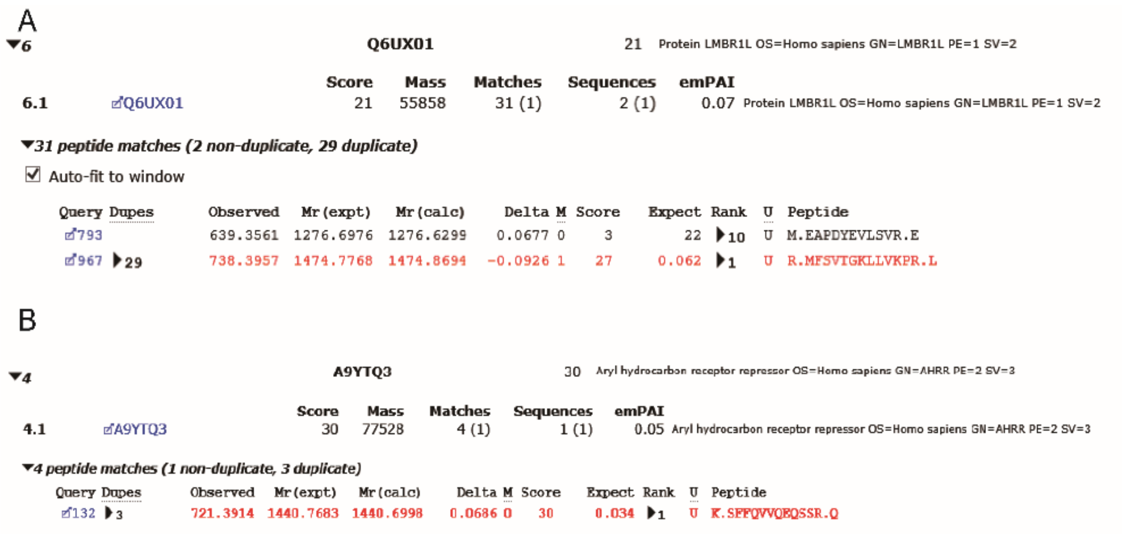

A

▼6 **Q6UX01** 21 Protein LMBR1L OS=Homo sapiens GN=LMBR1L PE=1 SV=2

		Score	Mass	Matches	Sequences	emPAI	
6.1	Q6UX01	21	55858	31 (1)	2 (1)	0.07	Protein LMBR1L OS=Homo sapiens GN=LMBR1L PE=1 SV=2

▼*31 peptide matches (2 non-duplicate, 29 duplicate)*

☑ Auto-fit to window

Query	Dupes	Observed	Mr(expt)	Mr(calc)	Delta	M	Score	Expect	Rank	U	Peptide
793		639.3561	1276.6976	1276.6299	0.0677	0	3	22	▶10	U	M.EAPDYEVLSVR.E
967	▶29	738.3957	1474.7768	1474.8694	-0.0926	1	27	0.062	▶1	U	R.MFSVIGKLLVKPR.L

B

▼4 **A9YTQ3** 30 Aryl hydrocarbon receptor repressor OS=Homo sapiens GN=AHRR PE=2 SV=3

		Score	Mass	Matches	Sequences	emPAI	
4.1	A9YTQ3	30	77528	4 (1)	1 (1)	0.05	Aryl hydrocarbon receptor repressor OS=Homo sapiens GN=AHRR PE=2 SV=3

▼*4 peptide matches (1 non-duplicate, 3 duplicate)*

Query	Dupes	Observed	Mr(expt)	Mr(calc)	Delta	M	Score	Expect	Rank	U	Peptide
132	▶3	721.3914	1440.7683	1440.6998	0.0686	0	30	0.034	▶1	U	K.SFFQVVQEQSSR.Q

Supplementary Figure 7. Mass spectrometry analysis of (A) the *LINC00305*-specific binding protein captured by a RNA pull-down assay in THP-1 cells and (B) the LIMR-interacting protein captured by a GST pull-down assay in THP-1 cells.

Figure. S8

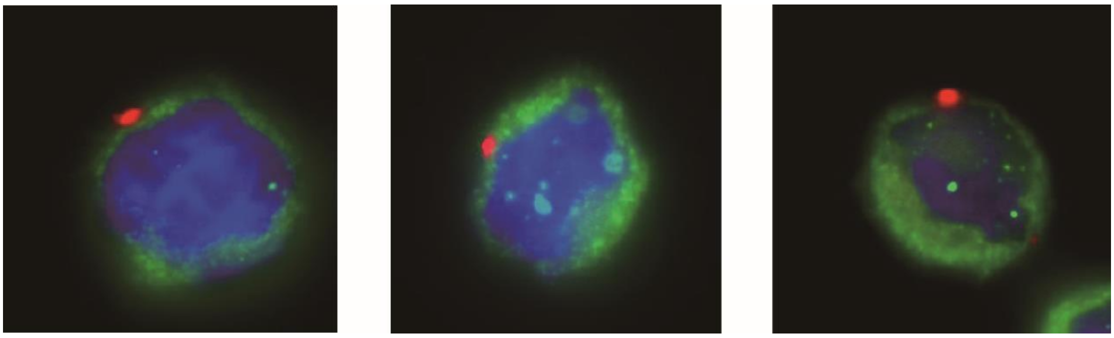

Supplementary Figure 8. *LINC00305* and LIMR co-localize in THP-1 cells. Representative images of THP-1 cells demonstrating the overlap of the *LINC00305* RNA-FISH signal (red) and the LIMR signal (green). DAPI staining of the cell nucleus is indicated in blue.

Figure. S9

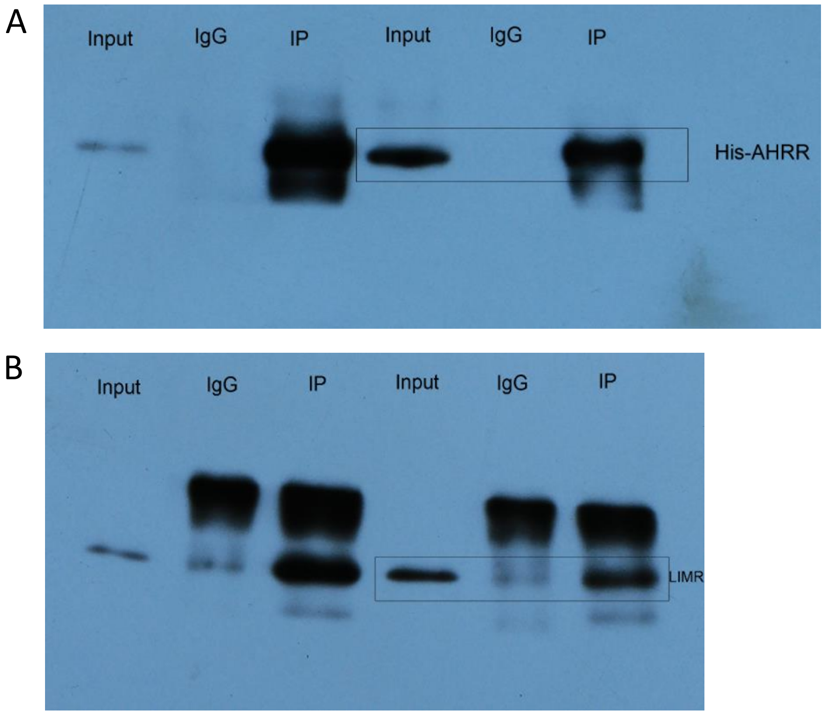

Supplementary Figure 9. The full-length blots to the co-IP analysis shown in Figure 5D. (A) Analysis with anti-His antibody. (B) Analysis with anti-LIMR antibody. For each antibody, two replicates of Input sample and the precipitated proteins were loaded. Rectangles indicate the cropped regions shown in Figure 5D.

Figure. S10

A

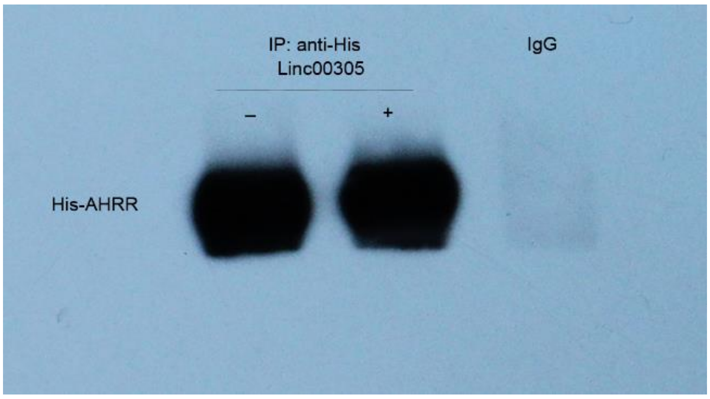

B

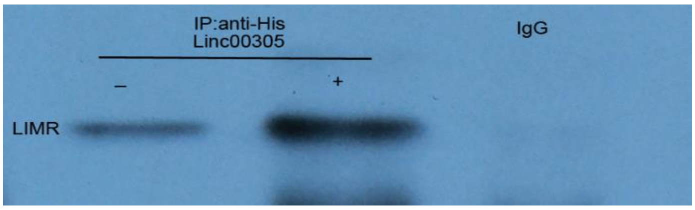

Supplementary Figure 10. The full-length blots to the co-IP analysis shown in Figure 5E. (A) **Analysis with anti-His antibody. (B) Analysis with anti-LIMR antibody.**
